# Factors influencing indicators of pregnancy and childbirth intention among female university students in South Korea: a cross-sectional study

**DOI:** 10.7586/jkbns.25.005

**Published:** 2025-02-21

**Authors:** Mi-Kyoung Cho, Gisoo Shin

**Affiliations:** 1 Department of Nursing Science, College of Medicine, Research Institute of Nursing Science, Chungbuk National University, Cheongju, Korea; 2 College of Nursing, Chung-Ang University, Seoul, Korea

**Keywords:** Stress, psychological, Universities, Students, Uncertainty

## Abstract

**Purpose:**

This study aimed to identify the factors influencing pregnancy and childbirth intention indicators among female university students in South Korea, where the lowest-worldwide fertility rate is a critical issue.

**Methods:**

This descriptive cross-sectional study was conducted among 646 female university students in Korea. The tools used in this study included measures of participants' demographic and physiological characteristics, pregnancy and childbirth intention indicators, right to self-determination, perceived stress, and intolerance of uncertainty.

**Results:**

Factors influencing pregnancy and childbirth intention indicators included age, expected pregnancy age, human papillomavirus (HPV) vaccination status, and self-determination, and the explanatory power of this model was 29.2%.

**Conclusion:**

Pregnancy and childbirth intention indicators were influenced by age, HPV vaccination, expected pregnancy age, and self-determination. These findings suggest that health-related factors such as HPV vaccination and the right to self-determination are critical determinants. Based on these results, this study provides evidence regarding the need for policy and educational interventions that support decision-making related to pregnancy and childbirth.

## INTRODUCTION

Recently, the issue of low birth rates has emerged not only in South Korea but also in countries including Europe, the United States, and East Asia [1]. The birth rate in South Korea has rapidly declined. As of March 2022, the total fertility rate was 0.86, the lowest in South Korean history and the lowest among the Organization for Economic Co-operation and Development countries [2]. Since 2001, the South Korean government has been implementing countermeasure policies in various fields by investing a large amount of budget to increase the total fertility rate, but to date, the results have been skeptical. Accordingly, the South Korean government has developed a new response strategy that focuses on improving the quality of life for women rather than simply aiming to increase the total fertility rate [3].

Improving the quality of life of individual women in connection with low fertility means that they will help women who have made the right to self-determination about pregnancy and childbirth to achieve successful pregnancy and childbirth [4,5]. This study's unique focus on self-determination and its influence on health behaviors, particularly in the context of reproductive health, significantly contributes to the field. Such self-determination has been shown to influence mental health aspects such as anxiety and depression in the context of sexual activity within marital relationships or with dating partners, as well as being associated with the frequency of risky sexual behaviors and violence. This highlights one perspective among various approaches related to sexual behavior [6]. However, individuals' self-determination is activated by environmental factors, such as stress or uncertainty [7].

Consequently, recent research has increasingly focused on elucidating the correlation between self-determination and health behaviors [8]. In broader domains, significant correlations were observed between self-determination and individual well-being, morbidity, and mortality rates [9]. It has also been reported to affect the onset and management of conditions such as alcohol consumption, smoking, obesity, diabetes, cardiovascular disease, heart attack, and stroke [10].

Additionally, one of the factors’ influencing self-determination is the quality of motivation, which can be categorized into intrinsic and extrinsic motivation [11]. Intrinsic motivation is characterized by engaging in behavior for its own sake. In contrast, extrinsic motivation involves performing actions for separable outcomes, such as specific rewards, social acceptance, proving something to oneself, or maintaining consistency between one's values and actions [12]. This type of motivation serves as a factor that sustains individual behavior's autonomy and significantly influences health behaviors [8-10]. Additionally, it serves as an indicator for predicting women's indicators for pregnancy and childbirth intentions (PCIs) [13].

Furthermore, the diversity of individual characteristics among women can influence PCI indicators. This diversity includes visible characteristics, such as race, age, education level, economic status, and residence, and invisible ones, such as values, preferences, perceived health status, stress, and uncertainty [14]. However, when using the PCI index as a predictive indicator, it should reflect the diverse characteristics of women.

Therefore, this study attempts to investigate how various characteristics, including self-determination, influence the PCI indicators of female college students in South Korea. The urgency and importance of this research lie in the fact that these findings can be utilized as indicators for women's PCI in the future and also highlight the need for further in-depth research in this area.

## METHODS

### 1. Study design

This study is a descriptive cross-sectional survey to identify factors influencing PCI indicators among female college students in South Korea.

### 2. Participants

The participants of this study were female undergraduate students enrolled in universities located in Seoul, Gyeonggi Province, Chungcheong Province, Gangwon Province, Jeolla Province, and Jeju Province. Participants were selected based on their agreement to participate after being informed about the purpose and methods of the study. The number of participants in the study was 620 people when calculated by applying linear multiple regression with a small effect size (f^2^) = .02, significance level (α) =.05, power (1-β) = .85, fixed model, R^2^ increase method, three tested predictors (right to self-determination, perceived stress, intolerance of uncertainty), and a total number of predictors 19 (including 16 participant characteristics) using the G*power version 3.1.9.6 program [15]. A total of 682 people were conducted, considering the dropout rate of about 10%.

### 3. Measurements

#### 1) PCI indicators

The PCI indicators were taken from a tool developed by Delgado [14] after approval to use the tool. The content validity of the modified tool was assessed using the Brislin back-translation method, which is effective for tool adaptation [16]. Subsequently, content validity was further validated through consultations with one physician specializing in women's health, two nursing administrators, and two nursing professors. A 5-point Likert scale was employed, and the content validity index was found to be .90. The tool consists of 22 items, with higher scores indicating stronger intentions for pregnancy and childbirth. This study confirmed reliability with a Cronbach's α of .88.

#### 2) Right to self-determination

The right to self-determination was assessed using the tool developed by Lee and Kim [17] after approval. The right to self-determination consists of 18 items on a 5-point Likert scale, with six items of autonomy, six of competence, and six of relatedness. The higher the total score, the higher the right to self-determination. The tool's reliability in this study was Cronbach’s α = .91.

#### 3) Perceived stress

Perceived stress was measured using the Korean version of the scale developed by Cohen et al. [18], which was validated by Lee and Lee [19] after approval to use the tool. Ten items were measured on a 5-point Likert scale, and the higher the total score, the higher the perceived stress. In this study, the reliability was Cronbach's α = .77.

#### 4) Intolerance of uncertainty

Intolerance of uncertainty was assessed using the tool validated by Noh [20] into a Korean version from the 12-item shortened version developed by Carleton et al. [21] after approval to use the tool. The intolerance of uncertainty tool consists of two sub-factors: seven items of prospective anxiety and five items of inhibitory anxiety. A 5-point Likert scale of 5 points was used, and the higher the total score, the higher the intolerance of uncertainty. The tool's reliability in this study was Cronbach’s α = .92.

### 4. Data collection

The data collection period for this study was between May 1 and July 30, 2018, and data collection was conducted in a non-contact state using the self-report questionnaire online survey method. The online responses to the questionnaire, voluntary consent to participate in the study, and explanation of the purpose of the study were presented to the participants, and the online survey was conducted only for those who understood and agreed to this survey.

### 5. Data analysis

This study analyzed 646 datasets from the collected data, excluding those with insufficient questionnaire content, using the SPSS version 28.0 (IBM Corp., Armonk, NY, USA) program. PCI indicators, the right to self-determination, perceived stress, and intolerance of uncertainty, which are individual characteristics and main variables of the participants, were analyzed with descriptive statistics of frequency, mean and standard deviation, and the difference between PCI indicators according to the characteristics of the participants was analyzed by independent t-test and one-way ANOVA, followed by post-test verification by Scheffé test. The correlation between each variable was analyzed with the Pearson correlation coefficient, and then the factors influencing PCI indicators were analyzed using a hierarchical multiple regression. The significance level of each statistic was adopted at *p* < .05.

### 6. Ethical considerations

Before conducting the study, approval was obtained from the Institutional Review Board (IRB No: 2017-101) of the Chung-Ang university affiliated with the researcher. Participants were informed about the study's purpose, duration, methods, and potential risks and benefits. They were assured that declining to participate or withdrawing consent after agreeing to participate would not result in any disadvantages. Additionally, participants were informed that they could withdraw from the study at any time, and any data associated with their withdrawal would be immediately discarded. As a token of appreciation, all participants in this study received a small gift.

## RESULTS

### 1. Participants characteristics

The average age of the participants in this study was 21.59 ± 3.25 years, with 436 participants (67.5%) aged between 21 and 24. A total of 247 participants (38.2%) reported a family monthly income of 4,000,000 KRW or more, 399 participants (61.8%) rated their health status as good, and 531 participants (82.2%) had a body mass index below 23 kg/m^2^. Most female college students, 390 participants (60.4%), reported exercising. Among the participants, 280 students (43.3%) reported having a sexual experience. The 489 participants (75.7%) indicated they were on a diet, 441 (68.3%) reported alcohol consumption, and 51 (7.9%) reported smoking. The expected age for marriage was 29.37 ± 2.94 years, while the expected age for pregnancy was 30.99 ± 2.90 years. The most significant number of participants, 184 (28.5%), attended universities in Gyeongsang Province (Table 1).

### 2. PCI indicators, right to self-determination, perceived stress, and intolerance of uncertainty

The mean score of participants' PCI indicators was 65.81 ± 12.13. The mean score of self-determination was 27.43 ± 4.39, the mean score of perceived stress was 29.07 ± 4.90, and the mean score of intolerance of uncertainty was 38.10 ± 7.02 (Table 2).

### 3. Differences in PCI indicators according to participants` characteristics

PCI indicators were higher in those over 21 (F = 7.01, *p* = .001) and in perceived good health status (F = 3.56, *p* = .029). Also, the PCI of the participants who exercised regularly was higher (t = −3.20, *p* = .001), and among the participants vaccinated, human papillomavirus (HPV) (t = −3.11, *p* = .002). Expected marriage age (t = 2.36, *p* = .019) and pregnancy age (t = 3.02, *p* = .003) showed statistically significant differences in PCI indicators. Regarding the place of residence, Gyeongsang-do showed the highest mean score, and Jeju-island showed the lowest mean score (F = 5.67, *p* < .001) (Table 3).

### 4. Correlation among PCI indicators, right to self-determination, perceived stress, and intolerance of uncertainty

PCI indicators showed a significant positive correlation with the right to self-determination (r = .41, *p* < .001) and intolerance of uncertainty (r = .48, *p* < .001), and PCI indicators showed a negative correlation with perceived stress (r = −.17, *p* < .001). Self-efficacy showed a significant negative correlation with perceived stress and intolerance of uncertainty (r = −.48, *p* < .001, r = −.30, *p* < .001). Perceived stress showed a significant negative correlation with intolerance of uncertainty (r = −.10, *p* = .014) (Table 4).

### 5. Factors influencing PCI indicators

To identify the factors influencing the PCI indicators of this study, when constructing a hierarchical model for personal characteristics, self-determination rights, perceived stress, and intolerance of uncertainty, variables showing differences in univariate analysis were entered into the model using the enter method. Variables were removed based on significance probability .10. Variables were selected based on significance probability .05, hierarchical multiple regression analysis was performed, and a regression model for PCI indicators was constructed. Therefore, each regression model for PCI indicators had a variance inflation index of 1.00 to 8.03, which was less than 10. The tolerance was 0.13 to 0.99, which satisfies more than 0.1, so there was no multicollinearity problem. Durbin-Watson was 1.95 ~ 2.02, close to 2, independent without autocorrelation, and the assumption of regression analysis was satisfied.

As a result, four factors influenced PCI indicators: age, HPV vaccination, expected pregnancy age, and the right to self-determination, and the explanatory power was 29.2% (Table 5).

## DISCUSSION

This cross-sectional survey study examined the factors influencing PCI indicators, focusing on their characteristics and targeting female university students in South Korea. Based on the results, this study discusses the influencing factors.

First, in this study, among the individual characteristics of the participants, it was found that age, expected age of pregnancy, and HPV vaccination influenced their PCI indicators.

The average age of the participants was 21.59 years old, and it was found that PCI indicators were higher in the group over 21 years of age, which can explain that it is a universal result as the interest in the opposite sex increases as the age increases. The number of heterosexual relationships increases [22]. In addition, the desired gestational age of the participants was 30.99 on average, and it was found that the PCI indicators were high in the group below the average age of hope for pregnancy. These results are inferred to be correlated with the average age of marriage desired by the participants. As of 2018, the average age of marriage for South Korean women was 30.4 years, and their actual first pregnancy average age was 31.9 years. Statistically, the first pregnancy occurs less than a year after marriage. Therefore, this result was similar to the result of reporting the desired age for first childbirth according to the average age of the participants [23].

Moreover, the results of this study showed that PCI indicators were higher in the group receiving HPV vaccination than in the non-receiving HPV vaccination group. Women with a heightened sense of self-determination are notably more likely to make decisions regarding contraception, sexual activity, and healthcare, which can directly impact reproductive health outcomes. Furthermore, women’s self-determination can influence their willingness to receive HPV vaccination, which holds significant importance as a preventive measure in reproductive health [24]. This is because women associate their sexual activity is a behavior with fertility and reproductive health [25].

Second, the right to self-determination showed statistically the highest explanatory power of the influence on the PCI indicators. Women's right to self-determination is a key factor in determining women's health in the life cycle and their quality of life. Women's right to self-determination includes the right to choose pregnancy and motherhood freely, i.e., number of babies, abortion status, time of delivery, and so on [26]. However, according to the World Health Organization, in 2024 [27], half of all pregnancies worldwide each year are unintended, which harms women's health, and it announced that women's right to self-determination in pregnancy and childbirth is limited. According to the result of a study, the rate of pregnancy and childbirth for women is determined by the decisions of their husbands or family, not the women's right to self-determination in India [28]. On the other hand, women wish to conceive and give childbirth according to their right to self-determination. Still, due to the insufficient socio-economic environment, there are some limitations in leading to actual practice [29]. This is associated with unwanted sexual behaviors or sexual violence against women and can act as a factor affecting women's physical and mental health [6,7].

Meanwhile, perceived stress and intolerance of uncertainty, which were suggested as major influencing factors on women's PCI indicators, were not statistically significant in the results of this study. This is suggested as a limitation of this study because it was conducted with only female university students. According to previous research [30], women who considered pregnancy and childbirth with a future-oriented perspective showed higher levels of self-efficacy or autonomy than those who had not. However, as this study targeted female college students, it is inferred that they may not have had the opportunity to consider pregnancy and childbirth yet profoundly. Therefore, as PCI indicators to be conducted, women's right to self-determination should be regarded, along with various environmental factors supporting it. This study was limited by its focus on female university students in South Korea and its lack of consideration for family, cultural, and socio-environmental factors influencing women's self-determination.

## CONCLUSION

This study aimed to identify the factors influencing the PCI indicators of female university students in six cities across South Korea. The results showed that age, expected pregnancy age, HPV vaccination, and self-determination were significant factors affecting the PCI of Korean female university students, with self-determination having the most important impact. However, as the study participants were limited to female university students, future research should expand the age range of participants to validate the findings and enhance the study's generalizability.

## Figures and Tables

**Table 1.** General and PCI-related Characteristics of Participants(N = 646)

Characteristics		n	(%)	M ± SD
Age (yr)	< 20	132	(20.4)	21.59 ± 3.25
	21~ 24	436	(67.5)	
	≥ 25	78	(12.1)	
Family monthly income (10^4^ won)^†^	< 200	68	(10.5)	
	200~ 299	145	(22.4)	
	300~ 399	185	(28.7)	
	≥ 400	247	(38.2)	
Perceived health status	Bad	51	(7.9)	
	Moderate	196	(30.3)	
	Good	399	(61.8)	
BMI (kg/m^2^)^†^	< 23	531	(82.2)	20.92 ± 2.83
	23.0~ 24.9	62	(9.6)	
	≥ 25	50	(7.7)	
Regular exercise	No	256	(39.6)	
	Yes	390	(60.4)	
Presence of chronic disease^†^	No	611	(94.6)	
	Yes	34	(5.3)	
Sexual experience	No	366	(56.7)	
	Yes	280	(43.3)	
STD history	No	484	(74.9)	
	Yes	162	(25.1)	
HPV vaccination	No	371	(57.4)	
	Yes	275	(42.6)	
Diet experience	No	157	(24.3)	
	Yes	489	(75.7)	
Alcohol drinking	No	205	(31.7)	
	Yes	441	(68.3)	
Smoking	No	595	(92.1)	
	Yes	51	(7.9)	
Expected marriage age (yr)^†^	< 29.37	340	(52.6)	29.37 ± 2.94
	≥ 29.37	288	(44.6)	
Expected pregnancy age (yr)^†^	< 30.99	320	(49.5)	30.99 ± 2.90
	≥30.99	299	(46.3)	
Residential area	Chungcheong-do	78	(12.1)	
	Gangwon-do	18	(2.8)	
	Gyeonggi-do	152	(23.5)	
	Gyeongsang-do	184	(28.5)	
	Jeju Island	4	(0.6)	
	Jeolla-do	101	(15.6)	
	Seoul	109	(16.9)	

PCI = Pregnancy and childbirth intention; M = Mean; SD = Standard deviation; BMI = Body mass index; STD = Sexually transmitted disease; HPV = Human papillomavirus.

† Missing value.

**Table 2.** Descriptive Statistics of the Variables (N = 646)

Variables	Items	M ± SD	Min ~ Max
PCI indicators	22	65.81 ± 12.13	28 ~ 107
Right to self-determination	18	27.43 ± 4.39	14 ~ 36
Perceived stress	10	29.07 ± 4.90	16 ~ 50
Intolerance of uncertainty	12	38.10 ± 7.02	14 ~ 60

M = Mean; SD = Standard deviation; Min = Minimum; Max = Maximum; PCI = Pregnancy and childbirth intention.

**Table 3.** Differences in PCI Indicators According to the Characteristics of the Participants (N = 646)

Characteristics		Pregnancy and childbirth intention			
		n	M ± SD	t or *F*	*p* (Scheffé)
Age (yr)	< 20^a^	132	62.73 ± 10.51	7.01	.001 (a < b)
	21~ 24^b^	436	66.20 ± 12.27		
	≥ 25^b^	78	68.83 ± 12.95		
Family monthly income (10^4^ won)^†^	< 200	68	68.47 ± 12.19	2.31	.075
	200~ 299	145	64.46 ± 10.99		
	300~ 399	185	66.78 ± 11.82		
	≥ 400	247	65.20 ± 12.85		
Perceived health status	Bad^a^	51	65.63 ± 12.67	3.56	.029 (a > b)
	Moderate^a^	196	63.94 ± 11.82		
	Good^b^	399	66.75 ± 12.14		
BMI (kg/m^2^)^†^	< 23	531	65.63 ± 11.69	2.30	.102
	23.0~ 24.9	62	64.40 ± 12.04		
	≥ 25	50	69.08 ± 16.24		
Regular exercise	No	256	63.94 ± 11.84	−3.20	.001
	Yes	390	67.03 ± 12.18		
Presence of chronic disease^†^	No	611	65.95 ± 12.03	1.15	.253
	Yes	34	63.50 ± 13.92		
Sexual experience	No	366	65.11 ± 11.39	−1.63	.104
	Yes	280	66.71 ± 13.01		
STD history	No	484	66.02 ± 11.64	0.78	.434
	Yes	162	65.16 ± 13.51		
HPV vaccination	No	371	64.53 ± 12.22	−3.11	.002
	Yes	275	67.52 ± 11.82		
Diet experience	No	157	64.82 ± 12.01	−1.18	.240
	Yes	489	66.12 ± 12.17		
Alcohol drinking	No	205	66.55 ± 11.46	1.06	.291
	Yes	441	65.46 ± 12.43		
Smoking	No	595	65.84 ± 11.96	0.27	.790
	Yes	51	65.37 ± 14.12		
Expected marriage age (yr)^†^	< 29.37	340	66.94 ± 12.04	2.36	.019
	≥ 29.37	288	64.68 ± 11.88		
Expected pregnancy age (yr)^†^	< 30.99	320	67.55 ± 11.66	3.02	.003
	≥ 30.99	299	64.69 ± 11.96		
Residential area	Chungcheong-do^a^	78	63.77 ± 13.02	5.67	< .001 (b > a)
	Gangwon-do^a^	18	64.28 ± 14.78		
	Gyeonggi-do^a^	152	63.59 ± 11.00		
	Gyeongsang-do^b^	184	69.42 ± 11.59		
	Jeju island^a^	4	56.00 ± 25.13		
	Jeolla-do^a^	101	67.42 ± 11.99		
	Seoul^a^	109	63.39 ± 11.48		

PCI = Pregnancy and childbirth intention; M = Mean; SD = Standard deviation; BMI = Body mass index; STD = Sexually transmitted disease; HPV = Human papillomavirus.

† Missing value.

**Table 4.** Correlations among the Variables (N = 646)

Variables	PCI indicators	Right to self-determination	Perceived stress	Intolerance of uncertainty
	r (*p*)			
Right to self-determination	.41 (< .001)	1	-	-
Perceived stress	−.17(< .001)	−.48 (< .001)	1	-
Intolerance of uncertainty	.48 (< .001)	−.30 (< .001)	−.10 (.014)	1

PCI = Pregnancy and childbirth intention.

**Table 5.** Factors Affecting PCI Indicators (N = 646)

Model	Variables		Model 1		Model 2		Model 3		Model 4	
			*β*	*t*	*β*	*t*	*β*	*t*	*β*	*t*
1	Age (Ref: < 20)	≥ 21	0.16	4.03^*^	0.21	5.05^*^	0.20	4.79^*^	0.18	4.44^*^
	Health status (Ref: Bad)	Good	−0.07	−1.06	−0.03	−0.48	−0.04	−0.56	−0.03	0.45
	Regular exercise (Ref: No)	Yes	0.06	0.85	0.07	0.90	0.05	0.68	0.03	0.40
	HPV vaccination (Ref: No)	Yes	0.12	3.00^*^	0.14	3.66^*^	0.13	3.40^*^	0.12	3.09^*^
	Expected marriage age (Ref: ≥ 29.37)	< 29.37	0.02	0.37	0.02	0.19	0.01	0.03	0.03	0.37
	Expected pregnancy age (Ref: ≥ 30.99)	< 30.99	−0.27	−2.29^*^	−0.20	−2.45^*^	−0.17	−2.09	−0.17^*^	−2.25^**^
	Residential area (Ref: Gangwon-do)	Gyeongsang-do	−0.12	−0.54	−0.10	−0.79	−0.08	−0.80	−0.05	−0.47
2	Perceived stress				−0.03	−0.56	−0.02	−0.41	−0.05	−0.59
3	Intolerance of uncertainty						0.01	0.13	0.01	1.10
4	Right to self-determination								.211	.026^**^
F (*p*)			7.44 (< .001)		7.76 (< .001)		6.44 (< .001)		9.08 (< .001)	
Adj. R^2 ^(%)			7.0		18.1		19.0		29.2	
Durbin-Watson			1.95		1.98		2.02		1.98	

PCI = Pregnancy and childbirth intention; Ref = Reference; HPV = Human papillomavirus; Adj = Adjusted.

*p < .05; **p < .001.

## Data Availability

Please get in touch with the corresponding author for data availability.
